# Female Genital Warts: Global Trends and Treatments

**DOI:** 10.1155/S1064744901000278

**Published:** 2001

**Authors:** Stanley A. Gall

**Affiliations:** Department of Obstetrics and Gynecology University of Louisville 550 South Jackson Street Louisville KY 40202 USA

## Abstract

The increasing incidence of human papillomavirus (HPV) infection and HPV-associated conditions such as genital warts in women is a global concern. Genital warts are a clinical manifestation of HPV types 6 and 11, and are estimated to affect 1% of sexually active adults aged between 15 and 49. HPV infection is also strongly associated with cervical cancer, and is prevalent in as many as 99% of cases. The psychological stress of having genital warts is often greater than the morbidity of the disease, and therefore successful treatment is crucial. Current treatments are patient-applied and provider-administered therapies. Imiquimod 5% cream, a patient-applied therapy, is an efficacious treatment with tolerable side-effects and a low recurrence rate, and has the potential to be an effective strategy for the management of genital warts.


Infect Dis Obstet Gynecol 2001;9:149–154

### Female genital warts: global trends and treatments

**Stanley A. Gall**

*Department of Obstetrics and Gynecology, University of Louisville, Louisville, KY*

The increasing incidence of human papillomavirus (HPV) infection and HPV-associated conditions such as genital warts in women is a global concern. Genital warts are a clinical manifestation of HPV types 6 and 11, and are estimated to affect 1% of sexually active adults aged between 15 and 49. HPV infection is also strongly associated with cervical cancer, and is prevalent in as many as 99% of cases. The psychological stress of having genital warts is often greater than the morbidity of the disease, and therefore successful treatment is crucial. Current treatments are patient-applied and provider-administered therapies. Imiquimod 5% cream, a patient-applied therapy, is an efficacious treatment with tolerable side-effects and a low recurrence rate, and has the potential to be an effective strategy for the management of genital warts.

Key words: HUMAN PAPILLOMAVIRUS; ANOGENITAL CANCER; PATIENT-APPLIED THERAPY; IMIQUIMOD

### PREVALENCE OF HUMAN PAPILLOMAVIRUS AND GENITAL WARTS

Human papillomavirus (HPV) infection is one of the three most common sexually transmitted diseases (STDs) in the United States, along with gonorrhea and chlamydia[1]. It is estimated that as many as 20–40% of sexually active women are infected with HPV[2]. In the US, the estimated number of new HPV infections each year is 5.5 million, with an estimated total prevalence of 20 million[3].

Over 100 different types of HPV exist, and they can be grouped according to their oncogenic potential (Table 1)[4]. HPV 6 and 11 are termed 'low risk', as they are rarely associated with carcinomas[5] and most commonly manifest as external genital warts[6]. Genital warts, however, have been found to be induced by other 'high' or 'intermediate' risk HPVs[5]. Genital warts are exophytic, confluent, cauliflower tumors and their typical morphologies aid their diagnosis. Their most common location in women is the vulva (Figure 1)[7].

**Table 1** Human papillomavirus (HPV) types and related oncogenic risk of cervical squamous carcinoma[4]

| *HPV type* | *Oncogenic potential* |
|---|---|
| 16, 18 | High risk |
| 31, 33, 35, 39, 45, 51, 52, 56, 58, 59, 68 | Intermediate risk |
| 6, 11, 26, 40, 42–44, 53–55, 62, 66 | Low risk |

The annual incidence of genital warts has increased steadily among women since the early 1950s, when the estimated incidence in the US was only 13 per 100 000 for the female population[8]. During the late 1970s genital wart prevalence increased to 106 per 100 000, and at present 1% of sexually active adults aged 15–49 are estimated to have genital warts[6]. The incidence of genital warts has also increased in Europe, with an approximately five-fold increase for females in the United Kingdom between 1971 and 1994[9], where they are

Correspondence to: Stanley A. Gall, MD, Department of Obstetrics and Gynecology, University of Louisville, 550 South Jackson Street, Louisville, KY 40202, USA. Email: sagall@louisville.edu

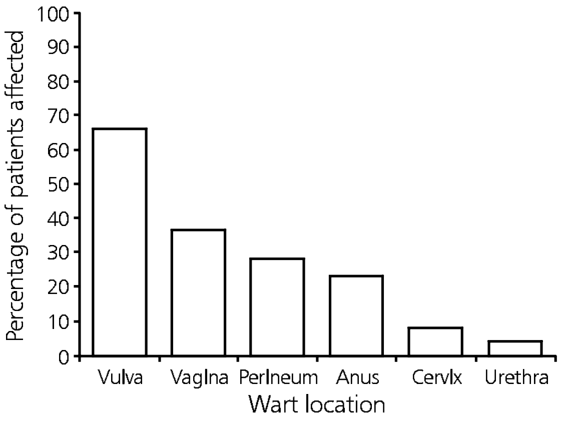

**Figure 1** Location of genital warts in women now the most commonly diagnosed STD in genitourinary medicine clinics[10].

### RISK FACTORS ASSOCIATED WITH HUMAN PAPILLOMAVIRUS INFECTIONS

Two factors that clearly influence the incidence of genital HPV infection are age and sexual behavior. As is the case with other sexually transmitted infections, prevalence is reported to be highest in sexually active young adults between 18 and 25 years of age[8]. Sexual behavior also has an influence on the potential risk of being infected with HPV. Women with five or more partners in the previous 5 years are over seven times more likely to have an episode of genital warts and are over 12 times more likely to have recurrent genital warts, compared with women with one sexual partner in this time[11]. Other risk factors reported in this study were a history of any other STDs, a history of oral herpes or a history of allergies[11]. There is conflicting evidence about whether smoking and use of oral contraceptives are also risk factors for genital warts[11–15].

Human immunodeficiency virus (HIV) infection is an additional determining factor for infection with other STDs, including genital warts. In one study, HIV-positive women were found to be over six times more likely to have genital warts, and over three times more likely to test positive for oncogenic HPV than HIV-negative women[16]. This study reported an annual incidence rate for genital warts in HIV-positive women of 11.4%, compared with 1.4% in HIV-negative women[16].

Furthermore, the host's immune status is also a factor that is important in controlling HPV infections and the development of HPV lesions such as genital warts. This is supported by the observation that patients who are immunosuppressed have an increased incidence of HPV-associated lesions[17]. Activation of the specific humoral and cellular immunity pathways is reported to be important in HPV-infected individuals for spontaneous regression of genital warts[18].

### HUMAN PAPILLOMAVIRUS AND ANOGENITAL CANCER

Although genital warts are considered to be a benign condition, an association between HPV infection and squamous cell carcinoma (SCC) of the anogenital tract has been identified[15]. Cervical cancer is the most common type of anogenital cancer; however, vulvar, vaginal and anal cancer are also associated with HPV[19]. HPV prevalence, in particular 'high risk' HPV types 16 and 18, is greater than 99% in cervical carcinomas[20]. Other HPV types that are associated with anogenital cancer are HPV 31, 33, 35 and 45 (Table 1)[21].

The increasing incidence of cervical intraepithelial neoplasias (CIN) in younger women[22] is of concern because of their malignant potential. For example, over 30% of CIN3 lesions progress to SCC within 1–10 years[22]. The progression from low-grade lesion to high-grade lesion or SCC is also reported to occur in approximately a third of cases[22]. The outcome of low-grade cervical neoplasia is influenced by a number of factors, including the oncogenicity of infecting HPV type, sexual behavior, infection of the cervical epithelium with other viral/bacterial agents, cigarette smoking, oral contraceptive usage and host immunosuppression[22].

### TRANSMISSION OF GENITAL WARTS

An important issue for women with genital warts is the concern about transmission of HPV and genital warts to their sexual partners. Transmission is believed to be predominantly through sexual intercourse, as genital HPV is absent in the majority of women who have not had sexual intercourse.

There have been studies, however, that have detected HPV DNA in cervical or vulva-vaginal samples from women who have not had sexual intercourse[8].

Transmission of HPV is enhanced when the superficial epithelium is disrupted[6], as this is where the infectious agent resides[4]. The HPV life cycle begins with infection of the basal cell layer of the epithelium and progresses with epithelial cell differentiation, resulting in complete virions present in the epithelial cells in the superficial layer[4]. The greatest risk of transmission is likely to exist when genital warts are present, as they reflect a productive HPV infection.

The risk of transmission of HPV to offspring is also a concern. The increasing frequency of childhood genital warts has been proposed to be the result of sexual abuse[5]. However, an association between genital warts and cutaneous HPV 2[23] has also been reported. There may also be a non-sexual transmission of genital warts from mothers, as well as the possibility of transmission during passage through the birth canal if mothers have external or cervical genital warts[5]. Additional data support a vertical (transplacental) transmission of HPV DNA, as over 50% of children born to HPV 16- or 18-infected mothers were positive for these HPVs[24].

### PSYCHOLOGICAL ASPECTS OF GENITAL WARTS

Genital warts are not only cosmetically unacceptable and associated with discomfort and pain, but they are also associated with emotional stress[25]. It is reported that the psychological stress of having genital warts is often greater than the medical effects of the disease[26]. Some of the psychological outcomes of patients with genital HPV infection are impairments to their sex life, a fear of cancer and a worsening of the emotional relationship with their partner[27].

In an international survey of patients' perceptions about genital warts it was found that 61% of women were 'quite' or 'very' concerned about having genital warts[25], with recurrence and transmission being of the greatest concern. In total, 95% of women believed that there was a risk associated with genital warts; the most commonly mentioned risks were a link to cervical cancer or to an unspecified cancer. In terms of lifestyle, approximately 40% of women said that having genital warts had changed their lifestyle. Sexual behavior had particularly changed, resulting in an increase in condom usage during sexual intercourse, abstinence from sexual intercourse, increased caution about new partners and a decrease in the number of sexual partners[25].

### TREATMENT OF GENITAL WARTS

Treatment of genital warts can be a frustrating experience for both physician and patients[28]. There are many current treatments for genital warts; however, none are successful in all three goals of therapy: complete eradication of warts, maintaining clearance and eliminating the virus. Recurrence is a substantial problem, as many therapies do not eradicate the reservoir of HPV DNA that is present in the tissue located adjacent to the genital wart.

Current therapies are composed of both ablative and cytodestructive modalities. Physically ablative therapies include cryotherapy, laser therapy, electrosurgery and surgical excision. Many of the physically ablative therapies have high initial success rates; however, recurrence rates are also high[28]. Cytotoxic agents that treat genital warts destroy the affected tissue either by chemodestructive or antiproliferative modes of action. Cytotoxic agents include podophyllin, podofilox (podophyllotoxin), trichloroacetic acid (TCA) and 5-fluorouracil.

Eradication of genital warts by stimulation of the immune system is an alternative strategy for genital wart therapies. Interferon, an antiviral, immunomodulatory agent is effective in treating genital warts when administered intralesionally[2], and can be used as a treatment for recalcitrant warts. Imiquimod is an immune response modifier that acts to induce both humoral (innate) and cellular immunity. The antiviral and antitumor activity of imiquimod is believed to be the result of the induction of cytokines such as interferon-$\alpha$, tumor necrosis factor-$\alpha$ and a number of specific interleukins[29]. It is this stimulation of the immune system to fight the HPV infection that is the proposed mechanism for eradication of genital warts by imiquimod.

In a pivotal clinical trial, complete clearance of lesions was observed in 72% of female patients treated with imiquimod 5% cream, three times a week for 16 weeks, or until warts cleared[28]. The response of a genital wart in a female patient to imiquimod treatment is shown in Figure 2. The low recurrence rate (13%)[29] in patients, an obstacle with many of the other current therapies, is an encouraging benefit of this treatment modality, and may reflect the development of specific long-term cell-mediated immunity.

Physicians have the choice between office-based or home-based therapies. The Centers for Disease Control and Prevention (CDC) guidelines for the treatment of genital warts recommend that the choice be guided by the preference of the patient, the available resources and the experience of the healthcare provider. It is recommended that providers are knowledgeable about, and have available to them, one patient-applied and one provider-administered treatment[21].

Numerous different factors can influence the choice of treatment by the physician. Treatment choice is based on morphology, number, distribution and the keratinization state of warts[30]. For example, soft non-keratinized warts respond well to podofilox and TCA, whereas keratinized lesions are better treated with physically ablative methods such as cryotherapy, excision or electrocautery[30]. Imiquimod is suitable for both types of lesions.

The choice of treatment should be influenced by the individual patient's preferences and convenience. Patient-applied treatments may suit patients who desire to have a less invasive form of treatment or more control over their care. They tend to be favored by patients owing to their convenience and ease of application.

Pregnancy is an important issue in the treatment choice for genital warts, especially as their number and size tend to increase during pregnancy[6]. It is advocated that genital warts are removed during pregnancy if causing complications[2]. Therapies such as cryotherapy, laser therapy and TCA may be used in the treatment of pregnant patients; however, the warts often spontaneously regress after delivery[5].

In addition, the cost of the treatment can influence the availability of a specific treatment choice. In a recent US pharmacoeconomic analysis of therapies, it was found that imiquimod is more cost-effective than podofilox as a first-line therapy, even though the average cost of treatment is lower for podofilox. This is due to the greater sustained clearance rate of imiquimod, compared with podofilox[31].

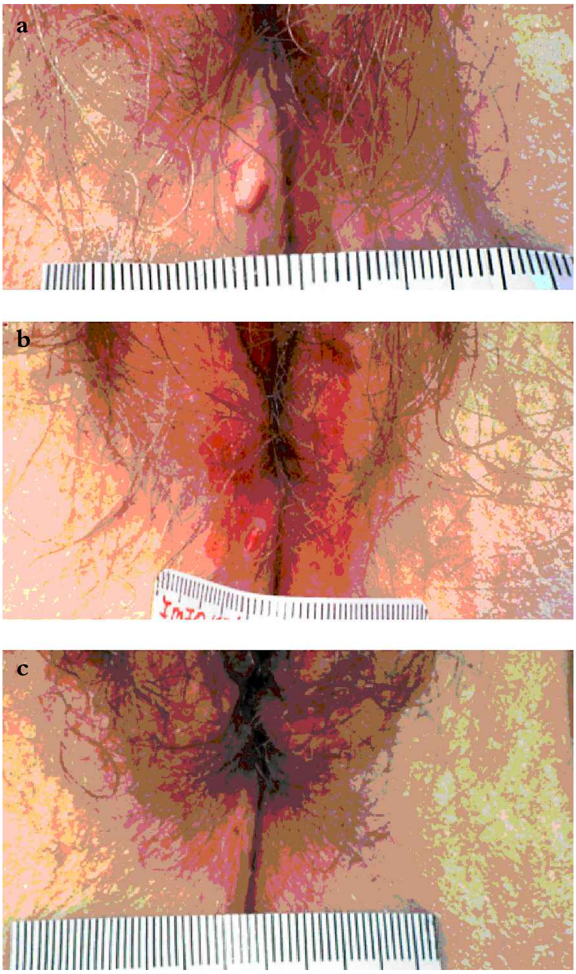

**Figure 2** Typical response of a genital wart in a female patient following treatment with imiquimod 5% cream. Photographs were taken at the beginning of treatment (a), after 6 weeks of imiquimod treatment (b) and 4 weeks after the end of treatment (c)

### KNOWLEDGE AND PREVENTION OF HUMAN PAPILLOMAVIRUS INFECTIONS

Studies of college students demonstrate a very low knowledge and awareness of HPV infections and genital warts[1]. Even though physicians provide information about genital warts[25], many patients are still misinformed and desire additional information. Informing adolescents and young adults about HPV and the risks associated with infection is an important consideration for the prevention of further increases in the incidence rates for HPV and genital warts. Aggressive health education strategies such as classes, magazine articles and internet information need to be employed to remedy this situation.

There is no simple method of preventing infection with HPV other than abstinence from sexual intercourse, an approach that has been shown to be relatively ineffective. HPV can be present in cells throughout the genital tract[1], and is transmitted by skin-to-skin contact. Barrier methods of birth control, which do reduce transmission, do not eliminate the possibility of infection[1]. A recent survey reported that approximately one-third of women attempt to prevent transmission of their genital warts by condom usage[25]. However, there is a risk of transmission from lesions that are not shielded by a condom[25]. It is still unknown at which stage patients with genital warts are infectious, and whether the elimination of the wart itself influences infectivity[2].

Prevention of cervical cancer relies primarily on the detection of intraepithelial disease through Pap smear screening[1], as opposed to the prevention of initial infection with HPV. The prospect of testing for HPV DNA in conjunction with Pap smears is currently under consideration[32].

### CONCLUSIONS

The increasing incidence of HPV infection and genital warts highlights the need for an effective strategy in the management of this disease. Even though many different treatment modalities exist, none of these have actually been proven to 'cure' the disease. New therapies such as imiquimod are improving therapeutic outcomes, but increasing public knowledge about prevention and transmission of HPV and genital warts is fundamental in the fight against these conditions.

### REFERENCES

1. Baer HS, Allen S, Braun L. Knowledge of human papillomavirus infection among young adult men and women: implications for health education and research. *J Community Health* 2000;25:67–78
2. Beutner KR, Wiley DJ, Douglas JM, *et al.* Genital warts and their treatment. *Clin Infect Dis* 1999; 28(Suppl 1):S37–56
3. Cates W. Estimates of the incidence and prevalence of sexually transmitted diseases in the United States. American Social Health Association Panel. *Sex Transm Dis* 1999;26(Suppl 4):S2–7
4. Cheah PL, Looi LM. Biology and pathological associations of the human papillomaviruses: a review. *Malays J Pathol* 1998;20:1–10
5. Jablonska S, Majewski S. Human papillomavirus infection in women. Special aspects of infectious diseases in women. *Clin Dermatol* 1997;15:67–79
6. Koutsky LA, Galloway DA, Holmes KK. Epidemiology of genital human papillomavirus infection. *Epidemiol Rev* 1988;10:122–63
7. Chuang TY, Perry HO, Kurland LT, Ilstrup DM. Condyloma acuminatum in Rochester, Minn, 1950–1978. II. Anaplasias and unfavorable outcomes. *Arch Dermatol* 1984;120:476–83
8. Schoultz DA, Koutsky LA, Galloway DA. Epidemiology and modes of transmission. In Gross G, von Krogh G, eds. *Human Papillomavirus Infections in Dermatovenereology*. Boca Raton, FL: CRC Press, 1997:83–97
9. Simms I, Fairley CK. Epidemiology of genital warts in England and Wales: 1971 to 1994. *Genitourin Med* 1997;73:365–7
10. Hughes GI, Simms I, Rogers PA, *et al.* New cases seen at genitourinary medicine clinics: England 1997. *Commun Dis Rep CDR* 1998;8(Suppl):S1–11
11. Habel LA, Van Den Eeden SK, Sherman KJ, *et al.* Risk factors for incident and recurrent condylomata acuminata among women. A population-based study. *Sex Transm Dis* 1998;25:285–92
12. Franco EL. Epidemiology of anogenital warts and cancer. *Obstet Gynecol Clin North Am* 1996;23: 597–623
13. Feldman JG, Chirgwin K, Dehovitz JA, Minkoff H. The association of smoking and risk of condyloma acuminatum in women. *Obstet Gynecol* 1997;89:346–50
14. Munk C, Svare EI, Poll P, *et al.* History of genital warts in 10 838 women 20 to 29 years of age from the general population. Risk factors and association with Papanicolaou smear history. *Sex Transm Dis* 1997;24:567–72

15. Jamison JH, Kaplan DW, Hamman R, *et al*. Spectrum of genital human papillomavirus infection in a female adolescent population. *Sex Transm Dis* 1995;22:236–43
16. Minkoff HL, Eisenberger-Matityahu D, Feldman J, *et al*. Prevalence and incidence of gynecologic disorders among women infected with human immunodeficiency virus. *Am J Obstet Gynecol* 1999;180:824–36
17. Petry KU, Scheffel D, Bode U, *et al*. Cellular immunodeficiency enhances the progression of human papillomavirus-associated cervical lesions. *Int J Cancer* 1994;57:836–40
18. Coleman N, Birley HD, Renton AM, *et al*. Immunological events in regressing genital warts. *Am J Clin Pathol* 1994;102:768–74
19. Stone KM. Human papillomavirus infection and genital warts: update on epidemiology and treatment. *Clin Infect Dis* 1995;20(Suppl 1):S91–7
20. Walboomers JMM, Jacobs MV, Manos MM, *et al*. Human papillomavirus is a necessary cause of invasive cervical cancer worldwide. *J Pathol* 1999; 189:12–19
21. Centers for Disease Control and Prevention. 1998 Guidelines for treatment of sexually transmitted diseases. *Morbid Mortal Weekly Rep* 1997; 47(RR-1):1–79
22. Handley J, Lawther H, Horner T, *et al*. Ten year follow-up study of women presenting to a genitourinary medicine clinic with anogenital warts. *Int J STD AIDS* 1992;3:28–32
23. Obalek S, Jablonska S, Favre M, *et al*. Condylomata acuminata in children: frequent association with human papillomaviruses responsible for cutaneous warts. *J Am Acad Dermatol* 1990; 23:205–13
24. Pakarian F, Kaye J, Cason J, *et al*. Cancer associated human papillomaviruses: perinatal transmission and persistence. *Br J Obstet Gynaecol* 1994;101:514–17
25. Maw RD, Reitano M, Roy M. An international survey of patients with genital warts: perceptions regarding treatment and impact on lifestyle. *Int J STD AIDS* 1998;9:571–8
26. Voog E, Lowhagen GB. Follow-up of men with genital papilloma virus infection. Psychosexual aspects. *Acta Dermatol Venereol* 1992;72:185–6
27. Filiberti AM, Tamburini M, Stefanon B, *et al*. Psychological aspects of genital human papillomavirus infection: a preliminary report. *J Psychosom Obstet Gynaecol* 1993;14:145–52
28. Beutner KR, Ferenczy A. Therapeutic approaches to genital warts. *Am J Med* 1997;102:28–37
29. Edwards L, Ferenczy A, Eron L, *et al*. Self-administered topical 5% imiquimod cream for external anogenital warts. *Arch Dermatol* 1998;134: 25–30
30. Maw R. UK National guidelines on sexually transmitted infections and closely related conditions. *Sex Trans Inf* 1999;75:S1
31. Langley PC, Richwald GA, Smith MH. Modeling the impact of treatment options in genital warts: patient-applied versus physician-administered therapies. *Clin Ther* 1999;21:2143–55
32. ALTS Group. Human papillomavirus testing for triage of women with cytologic evidence of low-grade squamous intraepithelial lesions: baseline data from a randomized trial. *J Natl Cancer Inst* 2000;92:397–402

RECEIVED 4/13/01; ACCEPTED 5/21/01
